# Improved Safety of Hybrid Electroconvulsive Therapy Compared With Standard Electroconvulsive Therapy in Patients With Major Depressive Disorder: A Randomized, Double-Blind, Parallel-Group Pilot Trial

**DOI:** 10.3389/fpsyt.2022.896018

**Published:** 2022-05-23

**Authors:** Jing-ya Zhang, Shu-xian Xu, Lun Zeng, Li-chang Chen, Jia Li, Zhao-yun Jiang, Bai-jian Tan, Chen-long Gu, Wen-tao Lai, Xiao-ming Kong, Jian Wang, Han Rong, Xin-hui Xie

**Affiliations:** ^1^Department of Clinical Psychology, Sleep Medicine Center, Second People’s Hospital of Huizhou, Huizhou, China; ^2^Department of Psychiatry, Renmin Hospital of Wuhan University, Wuhan, China; ^3^Department of Medical Statistics and Epidemiology, School of Public Health, Sun Yat-sen University, Guangzhou, China; ^4^Electroconvulsive Therapy Room, Department of Psychosomatic Medicine, Second People’s Hospital of Huizhou, Huizhou, China; ^5^Department of Psychiatry, Shenzhen Kangning Hospital, Shenzhen Mental Health Center, Shenzhen, China; ^6^Department of Psychiatry, Anhui Mental Health Center, Hefei, China; ^7^Brain Function and Psychosomatic Medicine Institute, Second People’s Hospital of Huizhou, Huizhou, China

**Keywords:** hybrid electroconvulsive therapy, electroconvulsive therapy, major depressive disorder, adverse event, cognitive function, double-blind, randomized, controlled trial

## Abstract

**Objectives:**

ECT is a rapid and effective treatment for depression. While efficacy is often remarkable over the initial 3–4 sessions, the efficacy of later sessions is less rapid, and the side-effects, especially cognitive impairment limit its use. To preliminarily compare the efficacy and acceptability of a novel hybrid-ECT (HECT) protocol for patients with major depressive disorder (MDD) with standard ECT, we conducted this pilot trial.

**Methods:**

Thirty patients were randomly assigned to ECT or HECT. Both arms received three ECT sessions (phase 1) but, in phase 2, the HECT arm received low-charge electrotherapy instead of ECT. The primary outcome was the change in 24-item Hamilton depression rating scale (HAMD-24) scores between baseline and the end of treatment. Cognitive function was assessed by repeatable battery for the assessment of neuropsychological status (RBANS), Stroop color word, and orientation recovery tests (ORT). Safety was measured by the drop-out rate and adverse events (AEs). Four visits were conducted at baseline, post-phase 1, post-phase 2, and at 1-month follow-up. Trial registration: Chinese Clinical Trial Registry (http://www.chictr.org.cn/), identifier: ChiCTR1900027701.

**Results:**

Patients in both arms showed significant within-group improvements in HAMD-24, but the between-group differences were non-significant. Participants in the HECT arm outperformed ECT patients for most cognitive tests at the end of treatment or at follow-up. There was a significantly lower AE rate and shorter ORT in phase 2 of the HECT ar.

**Conclusion:**

In this pilot trial, HECT was associated with fewer AEs and better cognitive function including executive and memory function, but its possible similar antidepressive efficacy needs to be further investigated in future.

## Introduction

Major depressive disorder (MDD) is a highly prevalent and debilitating mental illness, and associated with substantial disabilities. MDD patients often have a high suicide risk, severe impairments in social functioning, and cognitive dysfunction (1–3). In addition to personal suffering, depression is also related to significant distress and higher morbidity in family members and caregivers (4). Given the serious consequences of depression, a rapid antidepressive treatment is needed.

Electroconvulsive therapy (ECT) has been used in clinical practice for over 80 years and is widely considered the most effective acute antidepressant treatment (5). Nevertheless, its adverse events (AEs) (6, 7), including acute headache, dizziness and confusion, and impairment of cognitive function (8), limit its use. While the incidence of AEs is known to be affected by the stimulus intensity, number of treatments, and electrode placement (9, 10), achieving a rapid antidepressant effect while reducing AEs and cognitive deficits remains challenging.

Kellner et al. (11) conducted a double-blind, randomized controlled trial (RCT) to compare the efficacy and cognitive impact of three electrode placement methods in ECT and demonstrated that all placements resulted in rapid remission over the early course of treatment during the first 3–4 ECT sessions. Intriguingly, the slope of the Hamilton rating scale (24-item version, HAMD-24) score curve indicated that there was less therapeutic benefit from the later stages of ECT (after approximately 3–4 ECT sessions) than the first 3–4 ECT sessions. This observation suggested that fixed-charge ECT has different effects on patients at different stages of treatment, i.e., the marginal utility decreases with increasing number of sessions. Therefore, maintaining a consistently high electrical energy throughout the course of ECT may not be necessary. Furthermore, non-convulsive electrotherapy (NET), or low-charge electrotherapy (LCE), may have similar antidepressant effects to ECT but without serious AEs (12). Indeed, our own experience suggests that initial remission may be slower in patients receiving LCE than those receiving ECT. Therefore, it may be possible to exploit the benefits of ECT over the early stages of treatment but gain from sustained advantages of LCE over the longer term with fewer side-effects.

Therefore, we designed a simple but novel energy protocol for ECT, which we term hybrid-ECT (HECT) (13), in which the first three sessions use ECT for initial rapid remission and subsequent sessions use LCE to maintain the therapeutic benefits with minimal side-effects.

To test the efficacy and safety of HECT, we initiated two back-to-back preliminary randomized, double-blind, parallel-group, controlled clinical trials: one for schizophrenic patients and the other for MDD patients (reported here). The HECT RCT for schizophrenia completed several months earlier and demonstrated that, compared to ECT, HECT had similar antipsychotic effects but fewer AEs (14). Similarly, this trial tested the hypotheses that, compared to ECT (i) HECT exerts a similar antidepressant effect; and (ii) HECT causes fewer AEs and impairments in cognitive function, especially in the latter part of treatment (i.e., after three ECT sessions) and in the follow-up period.

## Materials and Methods

### Trial Information

This was a randomized, double-blind, parallel-group, standard-controlled pilot trial with a 1-month follow-up period. This single-center RCT was conducted at the Second People’s Hospital of Huizhou (Mental Health Center of Huizhou, Huizhou, Guangdong, China) in accordance with the Declaration of Helsinki (revised edition, 2008). The Human Ethics Committee of the Second People’s Hospital of Huizhou approved the study protocol (approval number: 2019-H04-01). Patients or their legal guardians could withdraw from the trial at any time for any reason. This trial was registered in the Chinese Clinical Trial Registry (see text footnote 1), registry number: ChiCTR1900027701 and is reported according to the CONSORT statement (15).

### Participants and Inclusion and Exclusion Criteria

Thirty MDD inpatients with or without psychotic features were recruited from December 1, 2019 to August 24, 2021. Eligible participants aged between 18 and 60 years or their legal guardians provided written informed consent. All patients met ICD-10 criteria for the diagnosis of major depression (16) using the Mini International Neuropsychiatric Interview (MINI) (17) and scored ≥ 21 on the HAMD-24 (18, 19). We excluded the patients if they: (1) failed to respond to earlier ECT; (2) had received ECT or repetitive transcranial magnetic stimulation (rTMS) over the previous 3 months; (3) had a diagnosis of bipolar disorder with or without psychotic features; (4) took medications incompatible with ECT treatment (such as lithium, benzodiazepines, antiepileptic drugs), with any use of these drugs stopped at least five half-lives before the start of ECT; (5) had a lifetime diagnosis of unstable, serious comorbidities (e.g., Parkinson’s disease, multiple sclerosis, stroke, alcohol use disorder) or history of epilepsy; (6) had < 9 years of education; (7) were pregnant or women without adequate contraception; and (8) were in other clinical studies or were unsuitable for participation as assessed by the investigators. All patients continued their usual antidepressive and psychotropic medications; no antidepressant pharmacotherapy changes were made during the treatment course.

### Electrode Placements

We used the bitemporal electrode placement, since bitemporal electrode placement achieved a faster earlier decrease in symptom ratings in a previous RCT (11). For this placement, the two electrodes were applied 2–3 cm above the midpoint of the line connecting the outer canthus of the eye and the external auditory meatus on each side of the head (20).

### Electroconvulsive Therapy/Low-Charge Electrotherapy Procedures

ECT/LCE procedures were standardized as follows: treatments were three times per week using a spECTRUM 5000Q ECT instrument (MECTA Corporation, OR, United States) with a pulse width of 1 ms and a fixed current of 800 mA. A dose titration procedure to determine seizure threshold was conducted at the first treatment following Mankad et al. (21), and a seizure duration (monitored by electroencephalography; EEG) > 15 s was considered successful (22, 23). Subsequent treatments were administered at 1.5-times seizure threshold for ECT sessions and, for LCE sessions, the energy was set at half the seizure threshold, regardless of whether seizures were induced. Anesthesia included administration of etomidate (0.2–0.4 mg/kg), muscle relaxation with succinylcholine (1–2 mg/kg) and atropine (0–1 mg) depending on heart rate, and ventilation with 100% O_2_ throughout the entire ECT or LCE procedures as described in our previous trial (14).

The decision to discontinue ECT/LCE was made by patient’s psychiatrist on the following basic principles based on the daily review: (1) relief of depressive symptoms; (2) insignificant benefits between the two recent ECT/LCEs; (3) side effects; and (4) other medical considerations.

### Interventions

The ECT arm was phase 1, three ECT sessions; phase 2, ECT for the remaining sessions. The HECT arm was phase 1, three ECT sessions (same as the ECT arm); phase 2, LCE for the remaining sessions (see Supplementary Figure 1).

### Pharmacotherapy

The individualized pharmacological regimen was determined by the patients’ psychiatrists. Patients in both arms maintained their previously prescribed antidepressants and antipsychotics (usually used for patients with depression and psychotic symptoms) during the trial. Anticonvulsant drugs, lithium, or mood stabilizers were discontinued during the ECT/LCE treatment. Single dose short half-life benzodiazepines were used as necessary when patients became agitated or felt anxious, but benzodiazepines were prohibited 24 h before ECT/LCE sessions. When patients suffered from insomnia, zopiclone, eszopiclone, or zolpidem were temporarily prescribed. Medication during the follow-up period was essentially the same as that during the ECT/HECT phase but with lithium, mood stabilizers, or antiepileptic drugs allowed.

### Randomization and Blinding

A 1:1 allocation sequence was generated using a random number generator^1^ by using a simple randomization method, and the sequence list remained concealed in opaque envelopes from the other researchers. The enrolled patients were assigned to the ECT or HECT arm according to this allocation sequence. Participants, the neuropsychological measurement rater, psychiatrists, nurses, and researchers were blinded to patient treatment assignment. To prevent the allocation information from being guessed based on the energy setting parameters on the ECT device, the ECT operator reset the parameters of the ECT equipment after each ECT/LCE session. Allocation status was concealed until the end of the follow-up period.

### Visit Schedule

The four visits were set at (1) baseline; (2) post-phase 1 (i.e., after three ECTs); (3) post-phase 2 (i.e., end of the treatment, within 24–48 h after the last ECT/LCE session); and (4) end of the 1-month follow-up period.

### Primary Outcome

The primary outcome was the change in HAMD-24 between baseline and end of the treatment (i.e., between visits 1 and 3). Response was defined as a decrease in total HAMD-24 score > 50% between baseline and end of treatment, and remission was defined as HAMD-24 total score < 8 at the end of the treatment.

### Secondary Outcomes

Changes between baseline and the end of the treatment in (1) Hamilton anxiety scale (HAMA) (24) and (2) positive and negative syndrome scale (PANSS) (25) were used to assess anxiety symptoms and psychiatric symptoms (only for patients with psychotic symptoms); (3) the everyday memory questionnaire (EMQ) (26) was used to measure subjective memory function and its impact on daily life at baseline and at the end of the follow-up period.

### Objective Cognitive Function Tests

The following cognitive function tests were used: (1) the twelve subtests of repeatable battery for the assessment of neuropsychological status (RBANS) (27) (list learning, story memory, figure copying, line orientation, picture naming, semantic fluency, digit span, coding, list recall, list recognition, story recall, and figure recall) were used to identify and characterize different aspects of changes in cognitive function; (2) the Stroop color word test was used as a measure of cognitive flexibility and control or executive function (28–30).

Orientation recovery tests (ORTs) after each ECT or LCE procedure were used to measure recovery in orientation after the patients finished the ECT/LCE session and were transferred to the recovery room. A trained nurse asked the patient the following five questions: “what’s your name?” “where are you?” “what’s the date today?” “how old are you?” and “when’s your birthday.” The ORT was recorded as the time point at which all five items were correctly answered.

### Safety Analyses

Any AE or patient who dropped out for any reason was recorded to analyze the safety of ECT/LCE.

### Assessment of the Effectiveness of the Blinding Methods

The rater and patients were asked to which arm they thought the participant had been allocated at the end of treatment and the end of follow-up period. The ratio of right/wrong guesses was used to evaluate the effectiveness of the blinding procedure.

### Sample Size

As this is the first HECT pilot study for MDD patients, we considered ethical, security, and statistical issues and set the sample number to 30 (15 participants per arm).

### Statistical Methods

For the main analysis, Welch’s two-sample *t*-tests were used for comparisons of the primary and secondary outcomes in the intention-to-treat (ITT) sample. Hedges’ *g* and its 95% confidence intervals (CI) were calculated to measure the effect size. For other ECT/LCE-related data such as anesthesia doses, seizure thresholds, number of sessions, and ECT/LCE parameters, Welch’s two-sample *t*-tests were used for comparisons of continuous outcome measures. For the safety results, Fisher’s exact tests were used to analysis differences in dropout and AE rates of different phases between the two arms. False discovery rate (FDR) correction (31) was conducted to accommodate false positive results. For the supplementary analysis, the generalized estimating equation (GEE) model was used to estimate the between- and within-group longitudinal trends in the primary, secondary, and cognitive outcome measures at four visits. The auto-regressive first order (AR1) was set as the working correlation matrix due to the characteristics of repeat-measure clinical data (32). All *p*-values less than 0.05 (two-sided) were considered significant. All statistical analyses were performed using R version 4.1.0 (R Project for Statistical Computing) within RStudio version 1.4.1106 (RStudio).

## Results

### Participant Flow

Fifty-eight inpatients were entered into the study; 28 screening failures were excluded after entry, and 30 patients were randomized into the ECT or HECT arms at a 1:1 ratio. The patient demographics at baseline are shown in Table 1. Due to symptoms, the baseline and post-phase 1 cognitive tests were not completed for one patient. One patient in each arm withdrew after phase 1 treatment and the results of their post-phase 1 visit were treated as the end of treatment visit for the primary and secondary outcomes according to the ITT principle; they also participated in the follow-up visits. Two patients from the ECT arm were lost to follow-up; all patients in the HECT arm completed follow-up visits. Due to the distance and the local COVID-19 prevention policy, face-to-face interviews at the follow-up visit were impossible with three patients, so they were interviewed online by video chats and, as a result, the cognitive tests were not performed. The details are presented in Supplementary Figure 1 and Supplementary Table 1.

**TABLE 1:** Baseline demographic and clinical characteristics.

Characteristics	ECT arm (*n* = 15)		HECT arm (*n* = 15)	
		
	*n*	*%*	*n*	*%*
Female	4	26.7	11	73.3
Psychotic symptoms	7	46.7	7	46.7
Suicidal ideation	15	100.0	15	100.0
Suicidal or self-injurious behavior	12	80.0	13	86.7
Treatment-resistant depression	8	53.3	9	60.0
Medication	15	100.0	15	100.0
SSRIs	8	53.3	7	46.7
SNRIs	7	46.7	6	40.0
Antipsychotics	9	60.0	9	60.0
	
	***Mean***	***s.d.***	***Mean***	***s.d.***
	
Age (years)	23.6	9.5	24.5	8.7
Education (years)	12.0	2.0	11.7	2.3
Onset of disease (years)	20.0	4.7	22.7	9.7
Disease course (years)	4.2	6.1	1.7	2.0
Current episode duration (months)	2.9	3.0	2.7	2.3
Equivalent fluoxetine dose (mg/day)	42.8	14.4	42.7	14.9
HAMD-24	34.3	7.6	34.5	7.8
HAMA	20.5	6.3	23.1	7.3
PANSS-P^a^	12.3	1.6	13.9	5.1
PANSS-N^a^	17.1	3.5	19.0	11.3
PANSS-G^a^	35.6	7.4	39.4	13.0
EMQ^b^	50.4	21.6	53.2	30.8

*^a^PANSS was assessed the patients who with psychotic symptoms, there were seven patients in each arm.*

*^b^One patient from the HECT arm did not complete the EMQ baseline evaluation.*

*ECT, electroconvulsive therapy; HECT, hybrid-ECT; SSRI, selective serotonin reuptake inhibitor; SNRI, serotonin and norepinephrine reuptake inhibitor; s.d., standard deviation; HAMD-24, Hamilton rating scale (the 24-item version); HAMA, Hamilton anxiety scale PANSS, positive and negative syndrome scale; PANSS-P, PANSS-positive scale; PANSS-N, PANSS- negative scale; PANSS-G, PANSS-general psychopathology scale; EMQ, everyday memory questionnaire.*

### Primary and Secondary Outcomes

In the ITT sample, the percentage decreases in HAMD-24 in the ECT and HECT arms were –68.3 and –59.14%, respectively. Thirteen patients in the ECT arm and 11 patients in the HECT arm reached response criteria (Fisher’s exact test, *p* = 0.651), and five patients in the ECT arm and four patients in the HECT reached remission criteria (Fisher’s exact test, *p* = 1.000).

**FIGURE 1:**
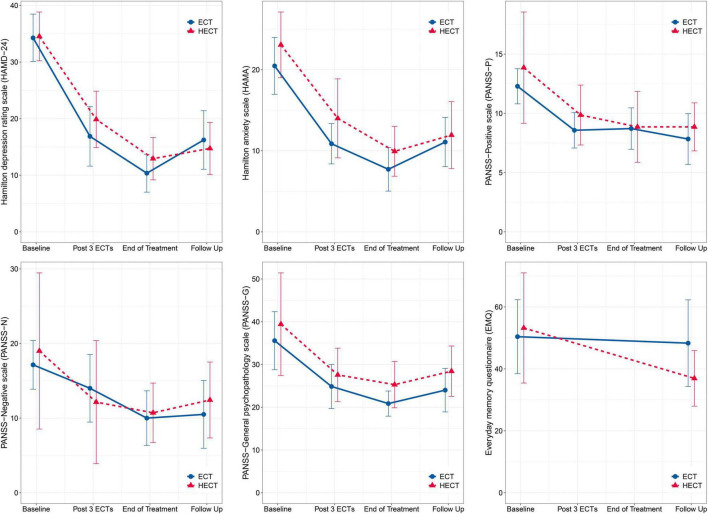
Changes in primary and secondary outcomes. There were no significant between-group nor group*time effects. The error bars represent 95% confidence intervals.

Changes in HAMD-24, HAMA, PANSS, and EMQ scores are shown in Figure 1, and detailed within- and between-group comparisons and effect sizes are shown in Table 2. Patients in both arms showed significant within-group improvements in HAMD-24, HAMA, and PANSS after treatment, but there were no significant between-group differences in these measures. For EMQ, the within- and between-group differences were both non-significant. In supplementary analyses, there were significant decreases in HAMD-24, HAMA, and PANSS in both arms, but there were no significant between-group nor group*time effects. The results of the supplementary analysis using the GEE model support the results of the main analysis and shown in Supplementary Table 2.

**TABLE 2:** Results of primary and secondary outcomes in the intent-to-treat (ITT) sample.

		ECT arm					HECT arm				Between-Group Effect			
					
	*n*	Pre- *mean (s.d.)*	Post- *mean (s.d.)*	Change *mean (s.d.)*	Heges’ *g (95% CI)*	*n*	Pre- *mean (s.d.)*	Post- *mean (s.d.)*	Change *mean (s.d.)*	Heges’ *g (95% CI)*	Heges’ *g* (95% CI)	*t*	*p*	*p.fdr* ^b^
**Primary outcome**														
HAMD-24	15	34.3 (7.6)	10.7 (5.8)	–23.5 (8.6)	–2.60 (–3.78, –1.58)	15	34.5 (7.8)	13.8 (7.1)	–20.7 (8.8)	–2.23 (–3.28, –1.32)	–0.31(–1.01, 0.39)	–0.884	0.384	0.898
**Secondary outcomes**														
HAMA	15	20.5 (6.3)	7.8 (4.5)	–12.7 (4.7)	–2.53 (–3.69, –1.53)	15	23.1 (7.3)	10.3 (5.4)	–12.7 (6.5)	–1.86 (–2.78, –1.05)	0.01 (–0.68, 0.71)	0.032	0.975	0.975
PANSS-P	7	12.3 (1.6)	8.7 (1.9)	–3.6 (2.5)	–1.24 (–2.33, –0.30)	7	13.9 (5.1)	8.9 (3.2)	–5.0 (4.1)	–1.06 (–2.07, –0.18)	0.39 (–0.60, 1.36)	0.789	0.449	0.898
PANSS-N	7	17.1 (3.5)	10.0 (4.0)	–7.1 (3.9)	–1.59 (–2.88, –0.52)	7	19.0 (11.3)	10.7 (4.3)	–8.3 (8.1)	–0.89 (–1.81, –0.06)	0.16 (–0.80, 1.12)	0.337	0.744	0.975
PANSS-G	7	35.6 (7.4)	20.9 (3.2)	–14.7 (7.4)	–1.72 (–3.07, –0.60)	7	39.4 (13.0)	25.3 (5.9)	–14.1 (10.0)	–1.23 (–2.32, –0.30)	–0.06 (–1.03, 0.92)	–0.122	0.905	0.975
EMQ	15	50.4 (21.6)	48.1 (21.4)	–2.3 (26.8)	–0.08 (–0.58, 0.41)	14^a^	53.2 (30.8)	37.9 (16.4)	–15.3 (27.3)	–0.53 (–1.09, 0.01)	0.47 (–0.26, 1.18)	1.294	0.207	0.898

*^a^One participant was excluded due to the lack of baseline EMQ data.*

*^b^False discovery rate (p.fdr) correction (Benjamini and Hochberg method). ECT, electroconvulsive therapy; HECT, hybrid-ECT; s.d., standard deviation; HAMD-24, Hamilton rating scale (the 24-item version); HAMA, Hamilton anxiety scale PANSS, positive and negative syndrome scale; PANSS-P, PANSS-positive scale; PANSS-N, PANSS- negative scale; PANSS-G, PANSS-general psychopathology scale; EMQ, everyday memory questionnaire.*

### Comparisons of General Electroconvulsive Therapy/Hybrid-Electroconvulsive Therapy Metrics and Safety Results

As shown in Table 3, ECT/LCE energy and seizure durations were significantly lower in phase 2 in the HECT arm than in the ECT group. Two participants withdrew during treatment after three ECTs: one patient from the HECT arm due to post-ECT headache, the other from the ECT arm for personal reasons. Two patients in the ECT arm were lost to follow-up for unspecified reasons. The drop-out rates were not significantly different between the two arms. As hypothesized, the differences in AE rates were not significantly different in phase 1 but were significantly lower in the HECT arm in phase 2.

**TABLE 3:** Comparisons of general metrics of ECT/HECT and safety results.

	ECT arm	HECT arm	*t*	*p*	*p.fdr* ^b^
Etomidate, mg, *mean (s.d.)*	25.73 (4.06)	23.20 (2.81)	1.987	0.058	0.174
Atropine, mg, *mean (s.d.)*	0.37 (0.28)	0.33 (0.24)	0.347	0.731	1.000
Succinylcholine, mg, *mean (s.d.)*	61.33 (11.26)	56.33 (7.12)	1.450	0.160	0.420
Seizure threshold, Joule, *mean (s.d.)*	14.40 (5.53)	12.35 (2.51)	1.306	0.207	0.483
**ECT/LCE number, *mean (s.d.)***					
Total	6.7 (1.7)	7.1 (2.0)	-0.602	0.552	0.966
Phase 1	3.0 (0.0)	3.0 (0.0)	0.000	1.000	1.000
Phase 2	4.0 (1.4)	4.4 (1.7)	-0.750	0.460	0.922
**ECT/LCE energy, Joule, *mean (s.d.)***					
Phase 1	20.4 (9.8)	16.8 (4.7)	2.244	0.028	0.099
Phase 2	23.0 (9.6)	6.8 (1.8)	12.417	<0.001	<0.001
**EEG seizure duration, s, *mean (s.d.)***					
Phase 1	63.2 (30.5)	61.4 (31.2)	0.277	0.783	1.000
Phase 2	40.5 (22.8)	14.3 (18.5)	6.807	<0.001	<0.001
**ORT, min, *mean (s.d.)***					
Phase 1	29.3 (9.2)	23.1 (6.5)	3.691	<0.001	0.002
Phase 2	30.1 (10.4)	16.2 (6.0)	8.772	<0.001	<0.001
**Safety**					
Withdrawal treatments, n/total patients	1/15	1/15	-	1.000^a^	1.000
Loss to follow up, n/total patients	2/15	0/15	-	0.483^a^	0.922
**AE, AE/non-AE after each session**			-		
Phase 1	17/28	18/27	-	1.000^a^	1.000
Phase 2	20/36	7/54	-	0.002^a^	0.009
**Blind effectiveness, guess right/wrong**					
**End of treatment**					
Patients	15/13		-	1.000^a^	1.000
Rater	15/13			1.000^a^	1.000
**End of follow up**					
Patients	15/13			1.000^a^	1.000
Rater	14/14		-	1.000^a^	1.000

*^a^Fisher exact tests.*

*^b^False discovery rate (p.fdr) correction (Benjamini and Hochberg method). ECT, electroconvulsive therapy; HECT, hybrid-ECT; LCE, low-charge electrotherapy; s.d., standard deviation; EEG, electroencephalogram; ORT, orientation recovery test; AE, adverse event.*

### Assessment of Blinding Effectiveness

As shown in Table 3, at visit 3, 15 of 28 allocation guesses made by patients and 15 of 28 made by raters were correct, while at visit 4, 15 of 28 allocation guesses made by patients and 14 of 28 made by raters were correct, which were not significantly different from the probability of random guessing. Thus, masking was successful for both patients and raters.

### Objective Cognitive Tests

**FIGURE 2:**
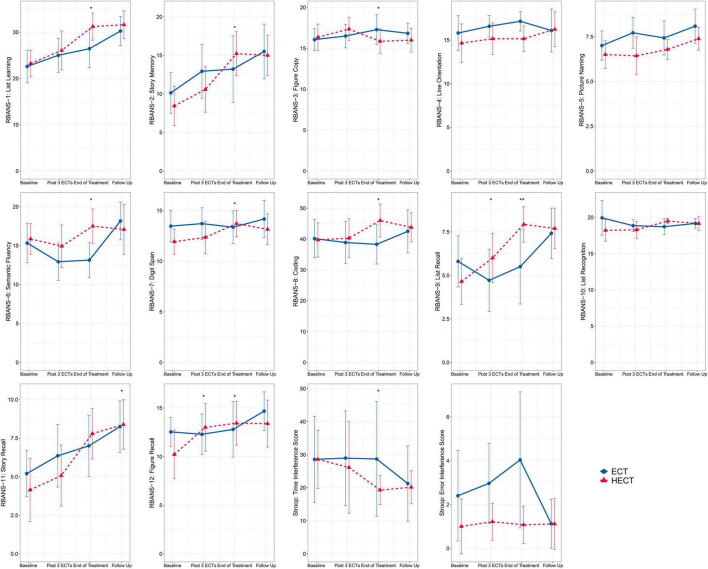
Changes in the twelve subtests of the repeatable battery for the assessment of neuropsychological status (RBANS) and the Stroop color word tests. The error bars represent the 95% confidence intervals, and asterisks mark significant group*time effects based on the generalized estimation equation (GEE) model: *p < 0.05, **p < 0.01.

As shown in Figure 2 and Supplementary Table 2, there were improvements in several cognitive measurements after treatments. At visit 3 (end of treatment) and 4 (follow-up), significant group*time effects were detected in eight RBANS subtests and Stroop time interference scores. With respect to RBANS subtests, there were significantly higher list learning, story memory, semantic fluency, digit spanning, coding, list recall, figure recall, and story recall scores in the HECT arm than in the ECT arm at visit 4. Conversely, only one RBANS subtest of figure copying was significantly lower in the HECT arm than in the ECT arm at visit 3. In terms of executive function, a significant lower Stroop time interference score was observed in the HECT arm than the ECT arm at visit 3 (end of treatment). Finally, a shorter ORT was observed in phase 2 in the HECT arm (Table 3).

In summary, participants in the HECT arm generally outperformed those in the ECT arm at the end of treatment for most cognitive tests.

### Data Accessibility

All individual participant data (IPD) are presented in Supplementary Table 1.

## Discussion

To our best knowledge, this is the first RCT of HECT for patients with MDD. Participants in both arms showed improvements in depressive symptoms after treatment, and no significant between-group differences in HAMD-24, HAMA, and three PANSS subscales were observed at all visits. In contrast, the HECT arm showed significantly fewer AEs and better cognitive function than those in the ECT arm during the second phase (i.e., LCE vs. ECT).

### Efficacy

HECT has two phases, the first consisting of three standard ECT sessions to achieve a rapid and immediate decline in depressive symptoms; the second containing several LCE sessions to reduce side-effects whilst maintaining efficacy. After phase 1, there was an ∼40% decrease in HAMD-24 from baseline, consistent with a previous study (11), with rapid remission induced by the first three ECTs demonstrated as expected. In phase 2, the decrease in HAMD-24 by LCE in the HECT arm was ∼20%, similar to the ECT arm. The moderate decline in symptom relief of LCEs was similar to that seen for ECT in the later stages of treatment, as predicted. Regarding whole treatment efficacy, the within-group mean effect sizes for the HAMD-24 in the ECT and HECT arms were approximately 2.60 and 2.23, respectively, consistent with previous RCTs of ECT (11, 33, 34). The between-group effect size of HAMD-24 was 0.31, which was non-significant, and the between-group difference remained non-significant at the end of follow-up.

In current ECT protocols, after the seizure threshold is obtained by titration or other empirical formulae such as the “half-age” strategy (35), the parameters of ECT generally remain the same throughout the course of treatment. This assumes that this high energy and its induced seizures are necessary for every ECT session throughout the course of treatment. However, this trial raises the prospect that high-energy ECT and its induced seizures may not be necessary in the later stages of ECT treatment.

### Acceptability and Safety

In ECT, it is as important to reduce side-effects as maintain anti-depressive efficacy. In this trial, HECT showed similar anti-depressive effects to ECT but a significantly better safety profile. There were no significant differences in AE rates in phase 1 treatment between the two arms, but the HECT arm showed significantly fewer AEs in phase 2 than the ECT arm, similar to our previous HECT trial in schizophrenia (14). The LCE energy in the present trial was set to half of seizure threshold, approximately 1/3 of the energy used in ECT. Although several seizures were induced during LCE sessions, patients in the HECT arm received lower energy, experienced significantly shorter seizure durations, and reported significantly fewer AEs in phase 2. Furthermore, other electrical treatments, such as rTMS and transcranial direct current stimulation, can also exert anti-depressive effects with fewer charge and side-effects (36, 37). Furthermore, the ORT was significantly shorter in the HECT group, as reported in our previous HECT trial (14). A study of magnetic seizure therapy (MST) found that the recovery times for consciousness were shorter in the MST group than ECT (38). MST also uses electrical currents to induce seizures; however, the strength of the electric field in the brain is smaller than ECT according to simulations in a realistic human head model (39). As the only difference between the HECT and ECT treatment is the much lower energy use after the third session, our trial indicates that the high energy use and its induced longer seizure durations in the later stages of ECT sequencing may further increase side-effects.

### Cognitive Function

The well-documented short-term side effects of ECT include retrograde amnesia and acute disorientation, which typically resolve within 3 days of treatment, as well as deficits in memory and executive function that resolve in days to less than 2 weeks after finishing an ECT course (8, 40, 41). In the present study, we used the EMQ to measure daily memory and found no significant change at baseline and follow-up in the ECT group, similar to previous studies on subjective memory (42, 43). Although there was a possible trend toward a better EMQ (lower scores) in the HECT group, the difference between groups was not significant, which may be due to the relatively small sample size and the high heterogeneity of the subjective memory test. We expect to increase the sample size in future studies.

In terms of objective cognition, participants in the HECT group outperformed those in the ECT group for most cognitive tests at the end of treatment. In both the RBANS and the Stroop tests, significant group*time interactions were mainly detected in executive function and objective memory, especially in short-term and working memory at visits 3 and 4. Previous studies have shown that executive and cognitive function recover with relief of depressive symptoms (40, 44, 45). In this study, both groups showed mild average improvements in cognitive and executive function after treatment, but cognitive improvements were more pronounced in the HECT group at visits 3 and 4. The current used in ECT is widely distributed in the hippocampus, amygdala, temporal, and frontal lobes (46) which are mainly associated with memory and executive function (47, 48). Conventional ECT with high currents or epilepsy tends to impact the prefrontal lobe related to executive function, whereas LCE with its lower current may reduce the associated executive impairment. Unfortunately, we were unable to determine whether the corresponding change was caused by a decrease in current or seizure duration. Distinguishing the separate roles of electric current and seizures in ECT will require new experimental paradigms to answer this question, which we are currently developing.

The hippocampus is an important brain region related to memory function (49), and hippocampal atrophy is one of the most reliable biomarkers of depressive disorders (50, 51). After ECT, the hippocampus—especially the neuronal cells of the dentate gyrus—grow significantly (neurogenesis) (52). In animal models, there is a dose-response effect between the increase in the number of new cells in the hippocampal dentate gyrus and the number of electroconvulsive seizures (ECS) (53). In reviewing physiological studies on epilepsy, we also found that neurogenesis is enhanced in the dentate gyrus after epileptic seizures but that the morphology of proliferating neurons is abnormal, such as the formation of basal dendrites in the dentate gyrus hilum and heterotrophic migration to the hilum (54). Seizures also lead to mossy budding of neurons present prior to the seizure. Epilepsy induces the proliferation of new neurons that can be integrated into the hippocampal circuit, but their fibrous connections are abnormal (54). The current study cannot establish whether the accumulated abnormal neurogenesis and immature nerve cells triggered by repeat seizures cause cognitive impairment, but the current study does suggest that lowering the energy during the later stages of ECT is beneficial to cognition, especially in terms of memory function.

### Limitations

This trial has several limitations. First, under ideal circumstances, a sham arm should be used as a placebo control group, but this is unethical in practice, so routine ECT was chosen as a standard control arm. Second, the between-group difference of antidepressive effects was non-significant, it may be just due to the relatively small sample size, but as a pilot RCT, the results demonstrated that HECT, as a new energy setting protocol, deserves further research. We expect to increase the sample size in future studies. Third, while several cognitive measurements improved after treatment, these may not necessarily have been due to the therapeutic effects of ECT or LCE, since the improvement in affective state may have secondarily improved the cognitive deficits associated with MDD (55–57). Instead, the between-group effects and the group*time effects may be more robust and relevant. Fourth, another major limitation is the short 1-month follow up period. Although there was no significant difference in response and remission rates between the two arms at the 1-month follow-up visit, HECT—as a “lower-energy” treatment—may be associated with a higher risk of relapse. Unfortunately, we were unable to answer this question, now. We expect to extend the follow-up period in the next trial to observe the long-term performance of HECT. Fifth, although the retrograde and autobiographical amnesia are the main cognitive impairments of ECT, considering the complexity and longtime cost of autobiographical memory test, we did not evaluate the autobiographical memory test in trial. We hope to rearrange memory-related tests to include the retrograde and autobiographical amnesia examinations in future studies. Finally, due to the coupling of electric current and seizures in ECT, it is very difficult to analyze the separate antidepressant (or other) effects of current and seizures, but we hope that our new research paradigm will overcome this problem.

## Conclusion

In the present pilot trial, HECT was found associated with fewer AEs and better cognitive function including executive and memory function. It also should be noted, notwithstanding that it failed to find a statistically significant difference in anti-depressive efficacy between groups, this result cannot be interpreted as the effects of the two arms definitely being the same. Nevertheless, HECT, as a novel and simple protocol, needs to be investigated in further trials.

## Data Availability Statement

The original contributions presented in the study are included in the article/Supplementary Material, further inquiries can be directed to the corresponding author/s.

## Ethics Statement

The studies involving human participants were reviewed and approved by the Human Ethics Committee of the Second People’s Hospital of Huizhou. The patients/participants provided their written informed consent to participate in this study.

## Author Contributions

J-YZ: investigation, data curation, and writing—original draft. S-XX: writing—original draft. LZ: investigation. L-CC: formal analysis and visualization. JL and Z-YJ: investigation and data curation. B-JT: supervision and funding acquisition. C-LG and W-TL: writing—review, editing, and formal analysis. X-MK and JW: writing—review and editing. HR: writing—review and editing, supervision, and funding acquisition. X-HX: conceptualization, methodology, formal analysis, visualization, supervision, project administration, writing—review and editing, and funding acquisition. All authors contributed to the article and approved the submitted version.

## Conflict of Interest

The authors declare that the research was conducted in the absence of any commercial or financial relationships that could be construed as a potential conflict of interest.

## Publisher’s Note

All claims expressed in this article are solely those of the authors and do not necessarily represent those of their affiliated organizations, or those of the publisher, the editors and the reviewers. Any product that may be evaluated in this article, or claim that may be made by its manufacturer, is not guaranteed or endorsed by the publisher.

## Footnotes

1
https://www.random.org/

## Supplementary Material

Supplementary Figure 1 Flow chart of the trial. ECT, electroconvulsive therapy; LCE, low-charge electrotherapy; HECT, hybrid-ECT; rTMS, repetitive transcranial magnetic stimulation.

Supplementary Table 1 Individual participant data.

Supplementary Table 2 Results of the supplementary analysis.
