# Supplementary figures and images for: Improved Safety of Hybrid Electroconvulsive Therapy Compared With Standard Electroconvulsive Therapy in Patients With Major Depressive Disorder: A Randomized, Double-Blind, Parallel-Group Pilot Trial
Source: Front Psychiatry. 2022 May 23;13:896018. doi: 10.3389/fpsyt.2022.896018 (PMC9168000; doi:10.3389/fpsyt.2022.896018)

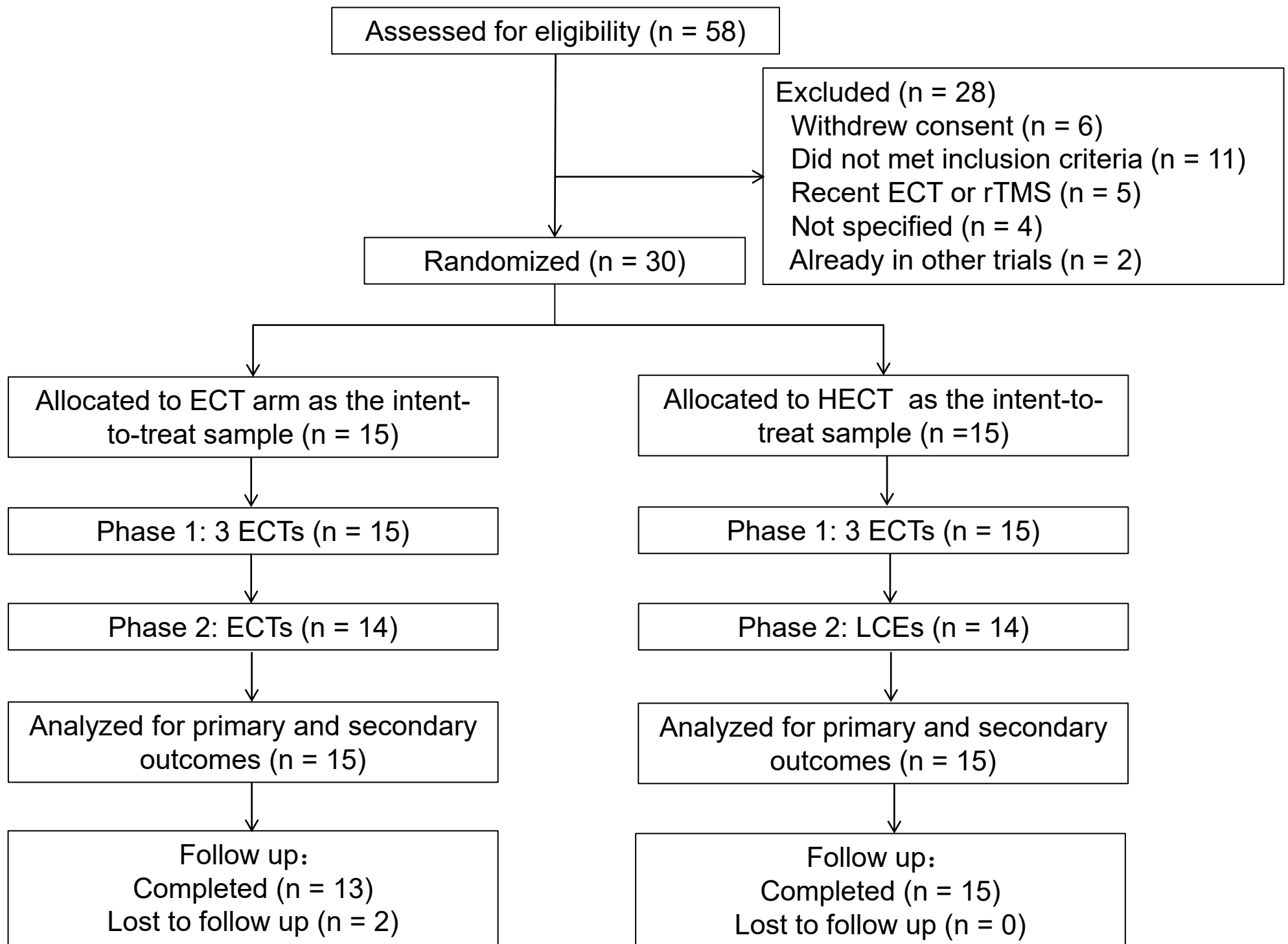

Supplement: Supplementary Figure 1 — Flow chart of the trial. ECT, electroconvulsive therapy; LCE, low-charge electrotherapy; HECT, hybrid-ECT; rTMS, repetitive transcranial magnetic stimulation. [file Presentation_1.PDF]
